# Characteristics, Ecological Risks, and the Impacts on Soil Carbon Cycling of PAH Pollution in the Soil of a Retired Coking Plant in Zaozhuang, Northern China

**DOI:** 10.3390/toxics14060503

**Published:** 2026-06-09

**Authors:** Liping Zheng, Yue He, Yifan Yan, Qun Li, Lei Zhang, Zhe Xing, Xiaosong Lu

**Affiliations:** Nanjing Institute of Environmental Sciences, Ministry of Ecology and Environment, Nanjing 210042, China; zlp@nies.org (L.Z.); yanyifan@nies.org (Y.Y.); liqun@nies.org (Q.L.); zhanglei@nies.org (L.Z.); xingzhe@nies.org (Z.X.); luxiaosong@nies.org (X.L.)

**Keywords:** PAHs, soil, coking plant, ecological risk, functional genes

## Abstract

During the industrial restructuring in China, numerous outdated coking enterprises were phased out. Despite the cessation of production for several years, the soil in the production area of the retired coking plant remains heavily contaminated with polycyclic aromatic hydrocarbons (PAHs), which continue to adversely affect soil health. However, research on the pollution characteristics of soil PAHs under prolonged PAH exposure and the associated changes in functional genes related to soil carbon cycling is still inadequate. This study aims to identify the pollution characteristics and ecological risks of PAHs in the coking plant and to investigate the effects of long-term PAH contamination from abandoned coking plants on the functional genes involved in soil carbon cycling. It was found that PAHs in the soil were predominantly composed of high-molecular-weight PAHs (HMW-PAHs), which constituted 65.7% to 83.4% of the total PAH content. The total concentration of PAHs in the surface soil ranged from 3.79 to 554 mg·kg^−1^, with an average concentration of 147.6 mg·kg^−1^. Source analysis based on isomer ratios indicated that PAHs primarily originated from the combustion of coal and biomass. Utilizing the toxicity equivalent factor (TEF) method, we found that the PAH levels in the CA group exceeded the Serious Risk Concentration, indicating that PAH pollution poses a potential threat to the ecological environment. Metagenomic analysis revealed that the gene abundance of alpha-amylase in the CA group was significantly higher than that in the OLA group (*p* < 0.05), suggesting that prolonged exposure to PAHs has enhanced the starch hydrolysis capabilities of soil microorganisms. The findings of this study refine methods for assessing the risks associated with soil PAH contamination and provide a theoretical foundation for the risk management and reuse of retired coking plant sites.

## 1. Introduction

Polycyclic aromatic hydrocarbons (PAHs) are a class of environmental organic compounds with multiple benzene rings. The environmental persistence and bioaccumulation of PAHs place them among the persistent organic pollutants (POPs) of greatest global concern [1]. Several PAHs (e.g., benzo[a]pyrene and dibenzo[a,h]anthracene) were classified by the International Agency for Research on Cancer (IARC) as Group 1 or Group 2 carcinogens [2]. For the toxins and high concentrations in the environment, 16 PAHs have been listed by the United States Environmental Protection Agency (U.S. EPA) as priority pollutants to control [3]. Sources of PAH pollution in the soil environment are widespread, with the diffusion and leakage of PAH-containing tar from coking plants, as well as combustion during industrial production, being among the major sources [4,5,6,7]. Years of coking plant production have caused severe PAH contamination in the soil. With rapid urbanization, many high-polluting industrial enterprises have been abandoned or relocated during the urban construction process. The soil pollution imparted by these sites has restricted land utilization. We must assess PAH contamination levels in the soil of retired coking sites and their subsequent ecological risks.

At the same time, microorganisms are highly sensitive to environmental changes, and soil microbial community changes can indicate the disturbance level of the soil environment via external factors [8,9]. Soil microorganisms mediate several important basic processes of the carbon cycle, such as carbon fixation, methane metabolism, and carbon degradation [10,11]. The microbial functions in biogeochemical processes depend on the activities associated with their functional genes [12]. PAHs in soil may disrupt normal biogeochemical processes, such as the carbon cycle, thereby affecting the ecological functions of the soil.

Long-term production activities at the coking plant have led to PAH contamination in the soil; this contamination may pose ecological risks, thereby limiting the soil’s potential for reuse. This study assessed a retired coking plant as its subject, examining the ecological risks of PAH contamination in the soil, and the soil carbon cycle functional genes were subjected to metagenomic analysis. In this study, we primarily focused on (I) exploring the pollution characteristics of PAHs in the coking plant’s soil; (II) assessing the ecological risk of PAHs in the soil; and (III) analyzing the impact of long-term PAH stress on the functional genes of soil carbon cycling. These findings refine our understanding of the ecological risks associated with PAH contamination at coking plants and their influence on the functional genes of the soil carbon cycle.

## 2. Materials and Methods

### 2.1. Study Area

**Figure 1:**
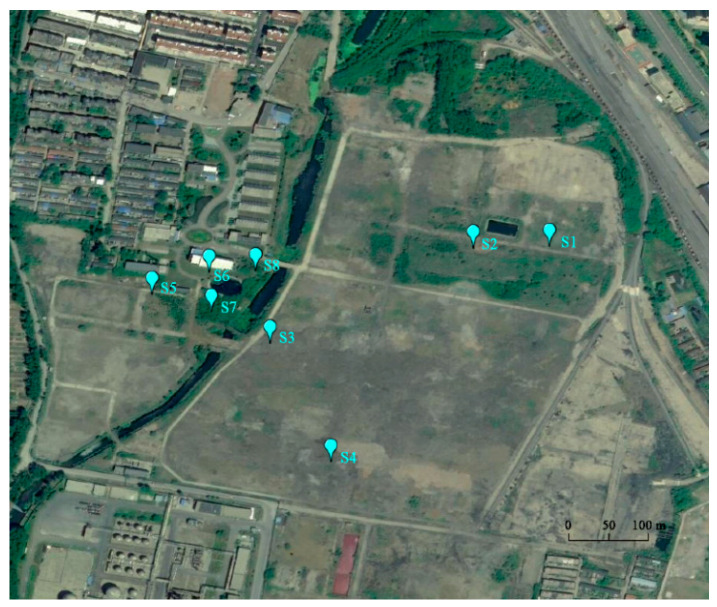
Remote sensing image of the study area.

Surface soil samples (0–20 cm depth) were collected at the site of a retired coking plant in Zaozhuang, a city in northern China. The study area is located in the mid-latitude warm temperate monsoon-type continental climate zone. The soil type is yellow-brown soil. Since its establishment in 1965, the factory has been engaged in coking production. Before the coking production, there was no pollution history. The area where the CA group is located is the production area of the coking plant. The production equipment and production activities of the coking plant are all located within the area of the CA group. The OLA group is the office area, with no coking production activities and no pollution sources. Sampling points were set, and samples were collected in the representative areas with heavy pollution, such as the coke oven, wastewater treatment, and tar storage area. Sampling was also conducted in the clean area of the office. The sampling points were established in different functional zones (Figure 1). Four sampling points (S1, S2, S3, S4) were established in the production area (CA group), and four sampling points (S5, S6, S7, S8) were established in the office area (OLA group), with three replicates at each point. Soil samples were collected in accordance with the HJ/T 166-2004 standard [13]. Using the sampling point as the center, a five-point sampling method was used to collect five samples within a 1 m × 1 m area, which were then mixed to form a composite sample. The samples were then sieved using a 2 mm diameter mesh to remove non-soil materials, such as stones or plant roots, and were stored in a 4 °C incubator. After the soil samples were collected, they were immediately transported to the laboratory for analysis.

### 2.2. Sample Analysis

Following the standard method (HJ 834-2017) [14], 16 types of PAHs prioritized for regulation by the U.S. Environmental Protection Agency (EPA) were extracted. Initially, 10 g of soil samples were weighed and mixed with silica sand to be ground into a fine powder. Then, the obtained samples were transferred to the accelerated solvent extraction (ASE) instrument (E-916, Stepka Laboratory Instrument Co., Ltd., Uster, Switzerland), where substitute standard substances (2-fluorophenol, 2-fluorobiphenyl, phenol-D6, nitrobenzene-D5, 2,4,6-tribromophenol, o-triphenyl-D14) were added, and a mixture of dichloromethane and acetone (1:1, *V*:*V*) was used for extraction. ASE extraction conditions: temperature was maintained at 100 °C; pressure was maintained at 100 bar; solvent was set as acetone/dichloromethane (1:1); preheating was maintained for 1 min; static extraction was conducted with pressure rising to 100 bar for 4 min; discharge time was set for 2 min; cycle was set as twice; solvent rinsing was set for 2 min; gas rinsing was set for 2 min. Following extraction and filtration, the sample was concentrated to 1 mL using a water bath, and a mixture of six deuterated polycyclic aromatic hydrocarbons was introduced as an internal standard. Subsequently, gas chromatography–mass spectrometry (7890B-5977B, Agilent Technologies Inc., Santa Clara, CA, USA) was employed to analyze the PAHs. Gas chromatography conditions were as follows: injection port temperature was 300 °C; injection volume was 1 μL; column flow rate was 1 mL/min; carrier gas was helium; column temperature was maintained at 45 °C for 2 min, then increased to 265 °C (at a rate of 20 °C/min), 285 °C (at a rate of 6 °C/min), and then 320 °C (at a rate of 10 °C/min), followed by maintenance for 4 min. For the electron impact source (EI), the following conditions were set: ion source temperature was 230 °C; interface temperature was 300 °C; fourth quadrupole temperature was 150 °C; mass scan range was 35–450 u; solvent delay time was 3 min; and data acquisition mode was set as full scan (Scan) mode. Qualitative analysis was conducted based on ion fragments and retention time, and quantitative analysis was performed using the quantitative ion internal standard method. The method’s detection limit was 0.1–0.2 mg/kg, the recovery rate ranged from 62% to 95%, and no blanks were detected.

### 2.3. DNA Extraction and Metagenomic Sequencing Analysis

The soil DNA extraction kit (DP712, Tiangen, Beijing, China) was used to disrupt the cell walls and membrane structures of microorganisms in the soil samples, thereby releasing the internal DNA. The extracted DNA fragments were sonicated into short DNA fragments of a specific length (350 bp), and adapter sequences were added to both ends to allow them to pair with the primers on the sequencer chip and undergo PCR amplification. The PCR products were then purified (AMPure XP, Beckman, Brea, CA, USA) and analyzed using a bioanalyzer (Agilent 2100, Agilent, Santa Clara, CA, USA) to analyze the size distribution of the library, followed by quantification of the library concentration using a Bio-Rad CFX96 instrument (Bio-Rad CFX96, Bio-Rad, Hercules, CA, USA). Index-coded samples were clustered using the cBot clustering system. After cluster generation, the library preparations were sequenced using a sequencer (NovaSeq 6000, Illumina, San Diego, CA, USA). The parameter for gene annotation is 1 × 10^−5^. The abundance of carbon cycle functional genes was uniformly standardized using TPM (Transcripts Per Million, transcripts per million reads). Metabolic (https://github.com/AnantharamanLab/METABOLIC, accessed on 3 June 2024) was used to annotate soil carbon functional genes and calculate their abundances. Shanghai Applied Protein Technology (Shanghai, China) conducted all analyses and quality control.

### 2.4. Ecological Risk Assessment

The Nemerow index method [15] is used to comprehensively assess the degree of soil contamination. The Nemerow pollution index (PN) is used to evaluate the PAH contamination level. Soil contamination can be classified into five levels: safe (PN ≤ 0.7), cautionary (0.7 < PN ≤ 1.0), slightly contaminated (1.0 < PN ≤ 2.0), moderate contamination (2.0 < PN ≤ 3.0), and severe contamination (PN > 3.0). The formula for calculating PN is as follows:

(1) $$P_{N}=\sqrt{\frac{p_{iave}^{2}+p_{imax}^{2}}{2}}$$

Pi represents the single-factor pollution index for contaminant i in the soil; pi = Ci/Si; Ci is the measured concentration of contaminant i; Si is the standard value for contaminant i, using the screening values for PAHs specified in GB 36600-2018 [16]; Piave is the average of all pollution indices in the soil; and Pimax is the maximum of all pollution indices in the soil. Due to the significant differences in toxicity levels among PAHs, the concentration of a single PAH cannot fully represent the overall risk of soil pollution. Therefore, the Nemerow index method (PN) is used to assess the comprehensive pollution degree of soil PAHs. The limitation of the Nemero index method lies in its tendency to amplify the maximum value, lack of weight differences, strong dependence on standards, and the resulting single comprehensive value. It has a weak ability to identify the spatial heterogeneity of regional pollution and is prone to evaluation errors. This evaluation method can be combined with single-factor analysis, such as the toxic equivalency quantity (TEQ) method, to enhance accuracy.

The BaP Toxic Equivalency Factor (TEF) was proposed by Nisbet and LaGoy in 1992 [17]. It is used to multiply the concentration of individual PAHs by a toxic equivalency coefficient to convert it to a BaP equivalent concentration, thereby assessing the ecological risk posed by PAHs. The formula for calculating the TEQ is as follows:

(2) TEQbap=∑Ci×TEFi

Ci represents the detected concentration of PAH component i, and TEFi represents the TEF value corresponding to component i. TEQbap represents the total toxicity equivalent of the 16 PAHs relative to BaP (mg·kg^−1^). Subsequently, the ecological risk of each group of soil samples was assessed based on the Serious Risk Concentration (SRC) for BaP of 7 mg·kg^−1^, as specified in the Dutch ecotoxicological models for environmental policy [18]. The toxicity equivalent method converts the mixture of multiple PAHs into a unified quantitative toxicity standard, effectively addressing the problem of significant differences in toxicity among different PAHs and the coexistence of various concentrations of PAHs in the soil for risk assessment. It is applicable to the ecological risk assessment of PAHs. The TEQ method assumes that the toxic effects of each component are additive, ignoring the synergistic or antagonistic interactions between chemical substances. This results in the actual risks being underestimated or overestimated. This method can only be used as a preliminary screening tool for specific pollution groups (such as polycyclic aromatic hydrocarbons and dioxin-like substances). When dealing with complex cross-category pollution, the TEF method cannot provide an accurate assessment of cumulative health risks.

### 2.5. Source Analysis

The isomers of PAHs exhibit the same migration, distribution, and dilution behaviors after entering the environment [19,20]. Generally, Low-Molecular-Weight PAHs (LMW-PAHs, with 2–3 rings) are derived from petroleum, while High-Molecular-Weight PAHs (HMW-PAHs, with 4–6 rings) are derived from combustion sources [21,22]. The isomer-ratio method was used to analyze the sources of 16 PAHs in the soil collected from the coking plant. Three commonly used PAHs ratios, BaA/(BaA + Chr), Flua/(Flua + Pyr), and Ant/(Ant + Phe), were used for the source analysis of PAHs in the soil.

### 2.6. Data Analysis

Origin 2021 (OriginLab, Northampton, MA, USA) and Excel 2020 (Microsoft, Redmond, DC, USA) were used to process the data. The R software (version 3.2.2) was used to carry out PCoA, Wilcoxon analyses, and Stacked bar chart. The R language packages are in the Supplementary Materials.

## 3. Results and Analysis

### 3.1. Content and Composition Characteristics of PAHs in Soil

The maximum total content of the 16 PAHs was 554 mg·kg^−1^, with an average of 147.6 mg·kg^−1^ (Table 1). The total content of seven carcinogenic PAHs (BaA, Chr, BbF, BkF, BaP, IcdP, DahA) was highest at Site S3 (275.2 mg·kg^−1^), accounting for 49.7% of the total PAH content at S3 (Table S1). Some studies have detailed high concentrations of soil PAHs in typical coking plant areas. Li investigated soil PAH contamination at a former coking plant wastewater treatment plant in Taiyuan, China [15], and found that the highest value of Σ16PAHs in the study area reached 1092.57 mg·kg^−1^. PAH analysis of soil samples collected from an abandoned coking plant (Beijing, China) by Cao indicated that, despite the plant having been abandoned for more than a decade, the degree of contamination remained severe [23]. The results of this study indicated that the soil samples from the CA group, which was the production area, exhibited typical characteristics of high PAH pollution concentration.

**Figure 2:**
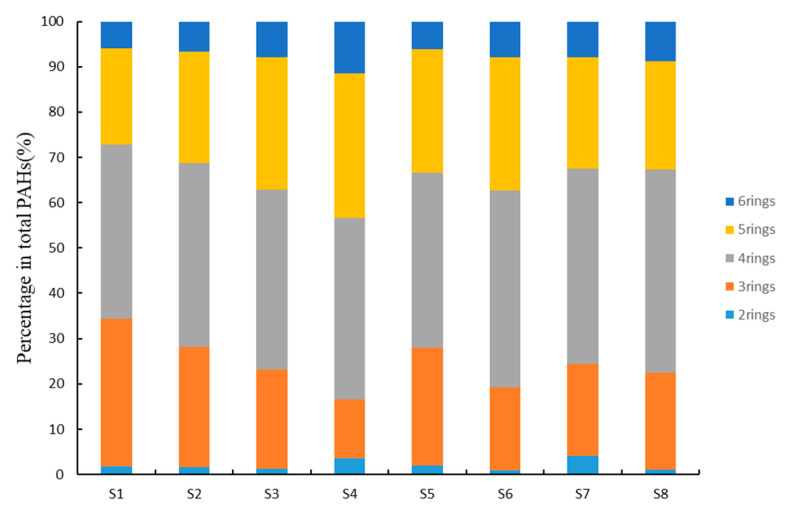
Proportion of different ring counts of PAHs in soil samples.

An analysis of the structural composition of PAHs in the soil samples (Figure 2) revealed that Low-Molecular-Weight PAHs (LMW-PAHs) accounted for a relatively low proportion (16.6–34.3%) in all soil samples, which were predominantly composed of High-Molecular-Weight PAHs (HMW-PAHs), ranging from 65.7% to 83.4%. Compared to LMW-PAHs, HMW-PAHs are composed of more benzene rings, have a more stable structure, and are less easily degradable. Although this coking plant has been abandoned for many years, the soil in the study area retains high levels of PAHs, with a high proportion of HMW-PAHs.

Low-ring PAHs (such as naphthalene and phenanthrene) have relatively high water solubility and may undergo environmental migration along with rainwater. High-ring PAHs (such as benzo[a]pyrene) have difficulty degrading in soil and have low water solubility. Li [15] conducted a PAH content analysis of surface soil at a retired coking wastewater treatment plant and found that high-ring types (5–6 rings) dominated, which is similar to the results of this study. High-ring PAHs (such as benzo[a]pyrene) have large molecular weights, difficulty dissolving in water, and high stability in the soil, thus mainly contributing to the soil PAH pollution at the coking plant with HMW-PAH characteristics.

### 3.2. Analysis of PAH Contamination Sources in Soil

**Figure 3:**
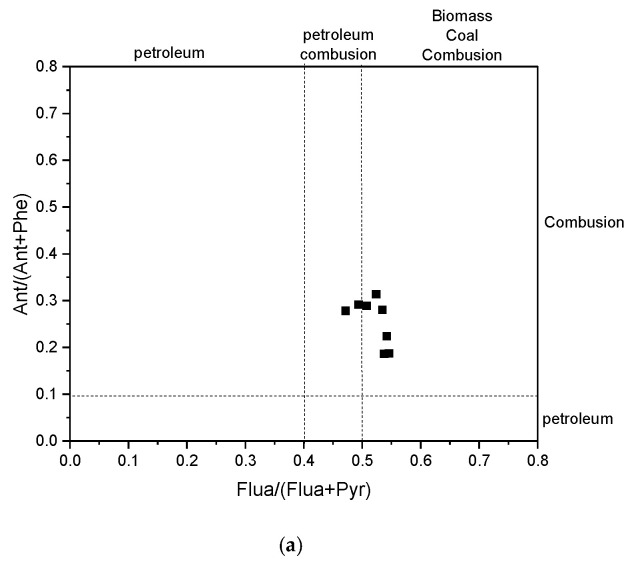

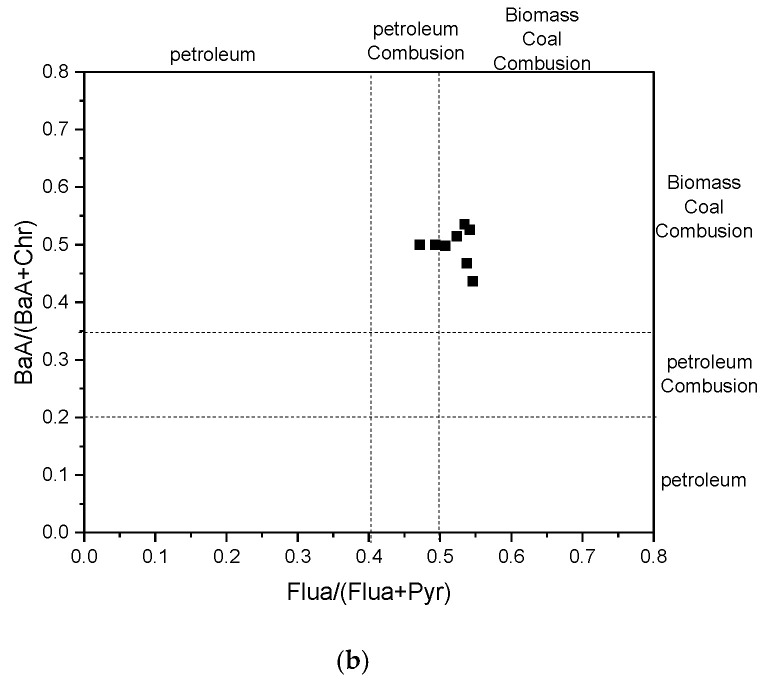
Source analysis of PAHs isomer ratio. (**a**) The ratio of PAH isomers based on Flua, Pyr, Ant and Phe; (**b**) The ratio of PAH isomers based on Flua, Pyr, BaA and Chr.

Source analysis of PAHs isomer ratio is shown in Figure 3. All the BaA/(BaA + Chr) values were greater than 0.35, ranging from 0.44 to 0.54, indicating that PAHs primarily originate from the combustion of coal and biomass. The 75% Fla/(Fla + Pyr) value is greater than 0.5 (S1, S2, S3, S4, S7, S8), ranging from 0.51 to 0.55, suggesting that the input of PAHs into the soil is primarily associated with coal and biomass combustion, with a portion originating from petroleum combustion. All of the Ant/(Ant + Phe) values were greater than 0.1, ranging from 0.19 to 0.31, indicating that all PAHs in the soil originate from combustion sources. Overall, the results indicate that PAHs in the soil primarily originate from coal and biomass combustion (S1–S4, S7, S8), with a portion originating from petroleum combustion (S5, S6). Fu [24] collected 10 surface soil samples from Linfen and conducted a source analysis, finding that coal combustion was the main source of PAHs in the soil; the findings of the study are consistent with our research. Due to the limited number of soil samples, there is some uncertainty in the source analysis of soil PAHs. PAH isomer ratios can be affected by weathering, degradation, transport, and aging in soil. The coking plant not only engages in coking production activities but also has human-driven vehicle transportation of goods. The exhaust gases emitted by car driving might cause partial pollution of the soil, which is caused by petroleum combustion. However, the majority of soil pollution might be caused by biomass and coal combustion during the coking production process, which leads to soil contamination by PAHs. During the urbanization process, due to human industrial activities, PAH contamination in some urban or suburban soils originates from the combustion of coal and biomass.

### 3.3. The Level of PAHs Pollution in the Soil

The Nemerow comprehensive pollution index (Table 2) showed that all the soils in the CA group (S1–S4) were severely polluted (PN > 3.0), while in the OLA group, the soils of S5 and S6 were moderately polluted (2.0 < PN ≤ 3.0), and the content of PAHs in S7 was at a safe level (PN ≤ 0.7). The single-factor index indicated that benzo(a)pyrene (BaP) was the PAH monomer with the highest pollution degree. The evaluation results showed that the PAHs in the soil of the CA group were severely polluted, and the degree of PAH pollution in this group was high. Due to the carcinogenicity of PAHs (such as benzo[a]pyrene), the long-term stress of PAHs in the soil of the coking plant may adversely affect the environment. During the coking production process, pollutants such as PAHs remain in the soil for a long time. The soil itself has a complex ecosystem, including soil animals, plants, and microorganisms. Soil pollution will have varying degrees of impact on all the organisms present in the soil. Compared with the screening values in the Soil Environmental Quality Construction Land Soil Contamination Risk Control Standards (Trial) (GB 36600-2018) [16], the concentrations of BbF, BaP, and DahA in the CA group exceeded the screening values. Many studies have included evaluations of abandoned industrial site soils, such as those found in steel plants, coking plants, and chemical plants [25,26]. A study on the soil after the relocation of a steel plant found that PAHs severely polluted the surface soil, and the Nemerow index of five pollutants, including benzo(a)pyrene, gradually decreased from the surface to the bottom, revealing the distribution pattern of pollutants in the vertical direction [27]. A study on the soil of a chemical plant also confirmed that there was severe organic pollution in the study area, and industrial sources were the main contributors [28].

### 3.4. Ecological Risk Assessment of PAHs in Soil

The TEQ_bap_ values for all soil samples ranged from 0.377 to 75.75 mg·kg^−1^, with the CA group ranging from 9.79 to 75.75 mg·kg^−1^ (Table S2). All values of the CA group exceeded the Dutch SRC threshold for ecological risk (7 mg·kg^−1^), indicating that PAHs in the CA group had a high ecological risk to the soil. The TEQ_bap_ values at all sampling sites in the OLA area ranged from 0.377 to 2.60 mg·kg^−1^, all of which were below 7 mg·kg^−1^, indicating a low ecological risk to the soil in the OLA area. The assessment results showed that PAHs in the CA group soil posed a high risk to the soil ecosystem, while the ecological risk of PAHs in the OLA group was low. The toxicity equivalents method was combined with the Dutch ecosystem-based Serious Risk Concentrations (SRCs) to identify that the CA group soil had a higher ecological risk. Some studies have included the use of the TEQ method to assess the ecological risk of soil PAHs. Li used the toxicity-equivalent method to evaluate the ecological risk of soil PAHs at a retired coking wastewater treatment plant in Taiyuan [15]. Meanwhile, Huang employed a combined approach of toxicity equivalents and risk quotients to assess the ecological risk of PAH-contaminated soil around oilfield wells [29]. The above research indicates that TEQ_bap_ is a widely used and reliable method for assessing the ecological risks of PAH-contaminated soil [30,31,32,33,34,35]. Since TEF is an estimation based on toxicological data, the assumption of mixed toxicity is dose additivity, and no consideration is given to synergistic and antagonistic effects. Therefore, the assessment results may have uncertainties. Hence, the assessment results are comprehensively evaluated using the Nemerow index method to determine the ecological toxicity of the soil.

### 3.5. Analysis of Genes Related to Soil Carbon Cycle

**Figure 4:**
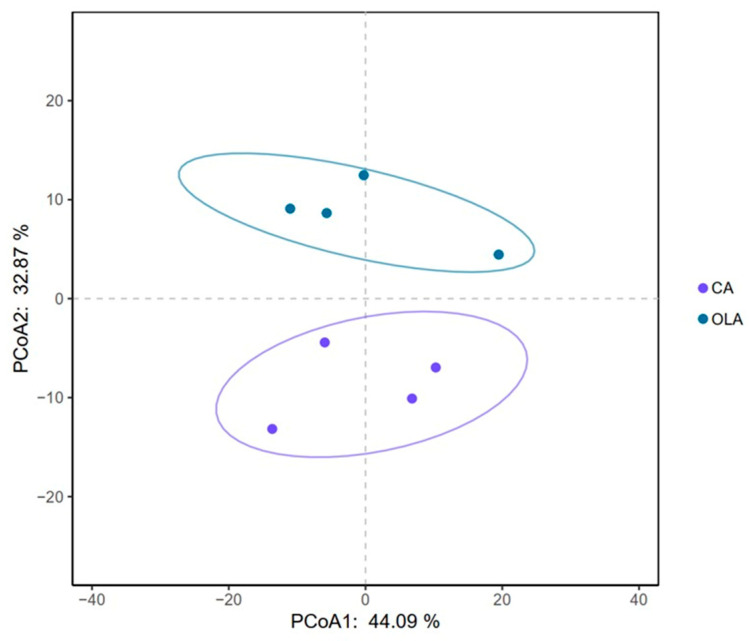
PCoA analysis diagram of genes of the carbon cycle.

Although OLA is located within the coking plant, it is situated in an unpolluted office area. Therefore, we compared the metagenomic results of the CA group with those of the OLA group. In order to analyze the differences in carbon cycle-related genes between the two groups, a PCoA analysis was performed (Figure 4). The results showed that the first principal component (PCoA1) accounted for 44.09% of the variance, while the second principal component (PCoA2) accounted for 32.87%. The CA group and the OLA group differed significantly along the second principal component, indicating that there are differences in carbon-cycle-related genes between the two groups.

**Figure 5:**
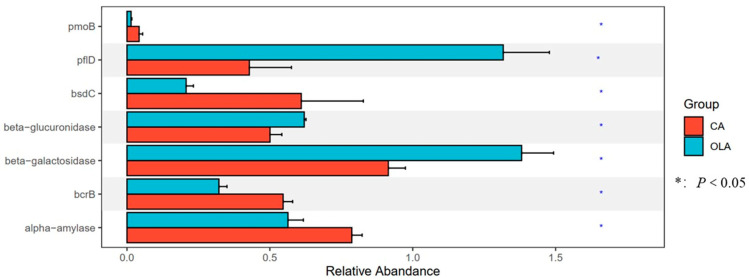
Significantly different functional genes.

We used the Wilcoxon test to identify the difference in functional genes between the CA and OLA groups; seven differentially expressed functional genes were identified (Figure 5). The analysis revealed that the abundance of the *pflD* gene in the OLA group was significantly higher than that in the CA group (*p* < 0.05). The *pflD* gene encodes pyruvate formate lyase (PFL). The significantly higher *pflD* gene abundance in the OLA group indicated that the OLA group possesses higher metabolic capacity regarding pyruvate metabolism in soil; long-term PAH stress may have inhibited pyruvate metabolism in the soil. The abundance of the alpha-amylase gene in the CA group was significantly higher than that in the OLA group (*p* < 0.05). Alpha-amylase primarily participates in the hydrolysis of starch; it catalyzes the hydrolysis of α-1,4-glycosidic bonds in starch to produce products such as glucose and maltose. Starch is one of the main storage forms of organic carbon in the soil. Through the action of α-amylase, organic carbon in the soil is decomposed to produce a large amount of small-molecule sugars (such as maltose and glucose). These small-molecule sugars are decomposed and utilized by microorganisms in the soil, and during the metabolic process, carbon dioxide is released through respiration, accelerating the soil carbon turnover. This is imperative to microbial carbon utilization [36]. The significant increase in alpha-amylase indicated that long-term PAH stress promoted the starch hydrolysis function of soil microorganisms and accelerated carbon utilization and metabolism in the soil [37,38,39].

## 4. Conclusions

In this study, we utilized a combined approach of risk assessment and metagenomics to investigate the ecological risks and impacts on soil carbon cycling associated with the retired coking plant’s soil. The soil contamination at the coking plant primarily consisted of high-molecular-weight polycyclic aromatic hydrocarbons (HMW-PAHs), which accounted for 65.7% to 83.4% of the total pollutants. The Nemerow index revealed that the soils in the CA group were severely polluted. The principal sources of soil PAHs were identified as coal and biomass combustion. Notably, the alpha-amylase gene in the CA group exhibited a significant increase (*p* < 0.05), suggesting that PAHs may enhance the starch-hydrolysis metabolism of soil microorganisms. This integrated methodology of ecological risk assessment and metagenomics provides a more comprehensive understanding of the environmental risks posed by the coking plant’s soil. It is imperative that retired coking plants implement remediation measures and effective environmental management strategies to mitigate the impact of PAHs on the environment. The limitations of this study include a restricted sampling quantity and background control; therefore, future research should aim to increase the sampling quantity to facilitate a more thorough investigation of various types of coking sites.

## Figures and Tables

**Table 1:** Statistics of the contents of 16 PAHs in surface soils of the coking plant (mg·kg^−1^).

PAHs	Min	Max	Mean	Std. Deviation	CV%	Sample Sizes
NaP	0.153	7.09	2.37	2.49	105.38	8
Acy	0.12	20.1	5.15	6.60	128.17	8
Ace	ND	7.7	2.13	2.59	121.77	8
Flu	0.12	11	4.05	4.79	118.31	8
Phe	0.433	59.8	17.84	21.62	121.21	8
Ant	0.1	27.4	7.05	9.36	132.79	8
Flua	0.6	68.4	19.65	23.62	120.16	8
Pyr	0.5	62.3	17.61	21.13	120.02	8
BaA	0.233	46	11.22	14.94	133.17	8
Chr	0.3	43.3	10.39	13.86	133.45	8
BbF	0.467	75.4	18.33	24.22	132.10	8
BkF	0.2	28.7	6.99	9.24	132.19	8
BaP	0.267	51.1	12.62	16.58	131.38	8
IcdP	0.15	24.3	5.97	7.79	130.51	8
DahA	ND	6.47	1.75	2.11	120.85	8
BghiP	0.15	19.5	4.99	6.22	124.87	8
Σ16PAHs	3.79	554	147.6			

Min: Minimum value; Max: Maximum value; Std. Deviation: Standard Deviation; CV%: coefficient of variation, CV (%) = standard deviation/mean × 100; ND: Not detected.

**Table 2:** Nemerow index (PN) of PAHs in soil samples.

	S1	S2	S3	S4	S5	S6	S7	S8
PN	17.37	32.88	66.72	8.62	2.26	2.31	0.35	1.31

## Data Availability

The original contributions presented in this study are included in the article/Supplementary Materials. Further inquiries can be directed to the corresponding author.

## Supplementary Materials

The following supporting information can be downloaded at https://www.mdpi.com/article/10.3390/toxics14060503/s1. Table S1: Concentration of 16 PAHs in each soil sample; Table S2: TEQ_bap_ of 16 PAHs in each soil sample.

## References

[1] You Q., Yan K., Yuan Z., Feng D.Y., Wang H.Z., Wu L.S., Xu J.M. (2024). Polycyclic aromatic hydrocarbons (PAHs) pollution and risk assessment of soils at contaminated sites in China over the past two decades. J. Clean. Prod..

[2] Fan K.Q., Feng Q.M., Li K., Lin J.Z., Wang W.D., Cao Y.B., Gai H.J., Song H.B., Huang T.T., Zhu Q.H. (2022). The metabolism of pyrene by a novel Altererythrobacter sp. with in-situ co-substrates: A mechanistic analysis based on pathway, genomics, and enzyme activity. Chemosphere.

[3] Karickhoff S. (1981). Semi-empirical estimation of sorption of hydrophobic pollutants on natural sediments and soils. Chemosphere.

[4] Xu S., Liu W., Tao S. (2006). Emission of polycyclic aromatic hydrocarbons in China. Environ. Sci. Technol..

[5] Peng C., Wang M., Zhao Y., Chen W. (2016). Distribution and risks of polycyclic aromatic hydrocarbons in suburban and rural soils of Beijing with various land uses. Environ. Monit. Assess..

[6] Lee R.G., Coleman P., Jones J.L., Jones K.C., Lohmann R. (2005). Emission factors and importance of PCDD/Fs, PCBs, PCNs, PAHs and PM10 from the domestic burning of coal and wood in the U.K. Environ. Sci. Technol..

[7] Li C., Zhang X., Wang X., Zhang X., Liu S., Yuan T., Qu W., Zhang Y. (2022). Distribution Characteristics and Potential Risks of Polycyclic Aromatic Hydrocarbon (PAH) Pollution at a Typical Industrial Legacy Site in Tianjin, North China. Land.

[8] Wang Y.C., Wu D.L., Liu J.X., Xu H.L. (2026). Polycyclic Aromatic Hydrocarbon Pollution Stress Impairs Soil Enzyme Activity and Microbial Community. Microorganisms.

[9] Wang C.Y., Wu H.T., Zhao W.N., Zhu B., Yang J.L. (2024). Effects of Polycyclic Aromatic Hydrocarbons on Soil Bacterial and Fungal Communities in Soils. Diversity.

[10] Umar Y., Sayed K., Syakir M.I., Al-Saedi T.K., Azhar B., Tohiran K.A., Nobilly F. (2025). Sustainable integrated oil-palm livestock practice to enhance soil organic matter and carbon sequestration potential. Int. J. Environ. Sci. Technol..

[11] Bardgett R.D., Freeman C., Ostle N.J. (2008). Microbial contributions to climate change through carbon cycle feedbacks. ISME J..

[12] Wang Q., Liu H.W., Jia S.X., Sheng J.G., Chen X.W., Zhang S.X., Zhang Y., Gao Y., Liang A.Z. (2023). Effect of conservation tillage on microbial functional genes related to carbon cycle of black soil. Acta Ecol. Sin..

[13] (2004). The Technical Specification for Soil Environmental Monitoring.

[14] (2017). Soil and Sediment-Determination of Semivolatile Organic Compounds-Gas Chromatography/Mass Spectrometry.

[15] Li Y., Zhang Q.X., Guo D.G., Dang J.H. (2023). Characteristics and Risk Assessment of PAH Pollution in Soil of a Retired Coking Wastewater Treatment Plant in Taiyuan, Northern China. Toxics.

[16] (2018). Soil environmental quality Risk control standard for soil contamination of development land.

[17] Nisbet I.C., LaGoy P.K. (1992). Toxic equivalency factors (TEFs) for polycyclic aromatic hydrocarbons (PAHs). Regul. Toxicol. Pharm..

[18] Posthuma L., Klok C., Vijver M.G., van den Brink P.J., van den Ende F., Traas T.P., Hendriks A.J. (2005). Ecotoxicological Models for Dutch Environmental Policy.

[19] Yunker M.B., Macdonald R.W., Goyette D., Paton D.W., Fowler B.R., Sullivan D., Boyd J. (1999). Natural and anthropogenic inputs of hydrocarbons to the Strait of Georgia. Sci. Total Environ..

[20] Yunker M.B., Macdonald R.W., Vingazan R., Mitchell R.H., Goyette D., Sylvestre S. (2002). PAHs in the Fraser river basin: A critical appraisal of PAHs ration as indicator of PAHs source and composition. Org. Geochem..

[21] Han J., Liang Y.S., Zhao B., Wang Y., Xing F.T., Qin L.B. (2019). Polycyclic aromatic hydrocarbon (PAHs) geographical distribution in China and their source, risk assessment analysis. Environ. Pollut..

[22] Zhang Q., Meng J., Su G., Liu Z., Wang T. (2021). Source apportionment and risk assessment for polycyclic aromatic hydrocarbons in soils at a typical coking plant. Ecotoxicol. Environ. Saf..

[23] Cao W., Geng S.Y., Zou J., Wang Y.Y., Guo Y.Q., Zhu Y., Dou J.F. (2020). Post relocation of industrial sites for decades: Ascertain sources and human risk assessment of soil polycyclic aromatic hydrocarbons. Ecotoxicol. Environ. Saf..

[24] Fu S., Cheng H.X., Liu Y.H., Xia X.J., Xu X.B. (2009). Composition, Distribution, and Characterization of Polycyclic Aromatic Hydrocarbons in Soil in Linfen, China. Bull. Environ. Contam. Toxicol..

[25] Tao S.Y., Ma J., Zhou Y.Z., Hou H., Zhao L., Sun J.L., Sun Z.J., Qin X.P. (2016). Polycyclic aromatic hydrocarbons pollution and risk assessment in soil of typical coal-fired pollution region in Shanxi Province. Ecol. Environ. Sci..

[26] Mohammed R., Zhang Z.F., Jiang C., Hu Y.H., Liu L.Y., Ma W.L., Song W.W., Nikolaev A., Kallenborn R., Li Y.F. (2021). Occurrence, Removal, and Mass Balance of Polycyclic Aromatic Hydrocarbons and Their Derivatives in Wastewater Treatment Plants in Northeast China. Toxics.

[27] Chen Y., Zhang J., Zhang F., Liu X.P., Zhou M. (2018). Contamination and health risk assessment of PAHs in farmland soils of the Yinma River Basin, China. Ecotoxicol. Environ. Saf..

[28] Geng S.Y., Cao W., Yuan J., Wang Y.Y., Guo Y.Q., Ding A.Z., Zhu Y., Dou J.F. (2020). Microbial diversity and co-occurrence patterns in deep soils contaminated by polycyclic aromatic hydrocarbons (PAHs). Ecotoxicol. Environ. Saf..

[29] Huang J., Zhou C., Song F., Li T.Y., Wang J.N., Fu X.W. (2025). Environmental DNA-Based Ecological Risk Assessment of PAHs in Aged Petroleum-Contaminated Soils. Toxics.

[30] Cao H., Li X., Qu C., Gao M., Cheng H., Ni N., Yao S., Bian Y., Gu C., Jiang X. (2022). Bioaccessibility and Toxicity Assessment of Polycyclic Aromatic Hydrocarbons in Two Contaminated Sites. Bull. Environ. Contam. Toxicol..

[31] Chai C., Cheng Q., Wu J., Zeng L., Chen Q., Zhu X., Ma D., Ge W. (2017). Contamination, source identifification, and risk assessment of polycyclic aromatic hydrocarbons in the soils of vegetable greenhouses in Shandong, China. Ecotoxicol. Environ. Saf..

[32] Wang Y.F., He L.S., Jiang D.L., Cao Y., Li Q., Gong J. (2021). Distribution and Ecological Risk Assessment of Polycyclic Aromatic Hydrocarbons and Heavy Metals in Coking Sites in China. Environ. Sci..

[33] Peng C., Wang M.E., Ouyang Z.Y., Jiao W.T., Chen W.P. (2012). Characterization and potential risks of polycyclic aromatic hydrocarbons in green space soils of educational areas in Beijing. Environ. Sci..

[34] Wang Q., Wang J., Cheng J., Zhu Y., Geng J., Wang X., Feng X., Hou H. (2023). A New Method for Ecological Risk Assessment of Combined Contaminated Soil. Toxics.

[35] Wang D., Ma J., Li H., Zhang X.C. (2018). Concentration and potential ecological risk of PAHs in different layers of soil in the petroleum-contaminated areas of the Loess Plateau, China. Int. J. Environ. Res. Public Health.

[36] Chia X.K., Hadibarata T., Risky A.K., Muhammad N.H.J., Inn S.T., Henry C.Y.F. (2024). The function of microbial enzymes in breaking down soil contaminated with pesticides: A review. Bioprocess Biosyst. Eng..

[37] Chia X.K., Hadibarata T., Muhammad N.H.J., Lies I.S., Inn S.T., Henry C.Y.F. (2025). Role of Extremophiles in Biodegradation of Emerging Pollutants. Top. Catal..

[38] Chia X.K., Hadibarata T., Muhammad N.H.J., Inn S.T., Henry C.Y.F. (2024). Extremophilic Microbes for Environmental Bioremediation: A Review. Biointerface Res. Appl. Chem..

[39] Lors C., Ryngaert A., Perie F., Diels L., Damidot D. (2010). Evolution of bacterial community during bioremediation of PAHs in a coal tar contaminated soil. Chemosphere.

