# Supplementary material for: Characteristics, Ecological Risks, and the Impacts on Soil Carbon Cycling of PAH Pollution in the Soil of a Retired Coking Plant in Zaozhuang, Northern China
Source: Toxics. 2026 Jun 9;14(6):503. doi: 10.3390/toxics14060503 (PMC13306952; doi:10.3390/toxics14060503)
Supplement: Supplementary file 1 [file toxics-14-00503-s001.zip › toxics-4299756-supplementary.pdf]

1. The R language packages

Stacked bar chart:

```
library(ggplot2)
library(dplyr)
library(tidyr)
```

PCoA :

```
library(phyloseq)
library(ggplot2)
library(vegan)
```

Wilcoxon:

```
library(ggplot2)
library(dplyr)
library(ggsignif)
```

2.

Table S1. Concentration of 16 PAHs in each soil sample($mg \cdot kg^{-1}$)

	S1	S2	S3	S4	S5	S6	S7	S8
NaP	3.35	5.18	7.09	2.43	0.4	0.16	0.153	0.16
Acy	6.65	10.3	20.1	2.22	0.88	0.58	0.12	0.34
Ace	7.7	2.73	3.4	0.17	0.6	0.2	ND	0.1
Flu	8.7	11	10.8	0.59	0.73	0.26	0.12	0.17
Phe	31.1	41.4	59.8	4	2.33	1.77	0.433	1.87
Ant	9	16.2	27.4	1.63	0.9	0.73	0.1	0.43
Flua	28.2	44.6	68.4	8.53	2.2	2.53	0.6	2.17
Pyr	23.9	38.9	62.3	8.3	2.47	2.6	0.5	1.87
BaA	11.9	22.5	46	4.87	1.67	1.63	0.233	0.97
Chr	10.7	19.5	43.3	4.9	1.67	1.63	0.3	1.1
BbF	19	34.9	75.4	10	2.73	2.67	0.467	1.5
BkF	7.27	13.5	28.7	3.67	1	1	0.2	0.6
BaP	13.3	25.2	51.1	6.6	1.73	1.77	0.267	1
IcdP	6.2	11.2	24.3	3.87	0.67	0.77	0.15	0.6
DahA	1.6	2.73	6.47	0.87	0.23	0.2	ND	0.15
BghiP	5.3	9.23	19.5	3.73	0.6	0.77	0.15	0.6
∑PAHs	194	309	554	66.4	20.8	19.3	3.79	13.6

3.

Table S2. TEQ_{bap} of 16 PAHs in each soil sample

	TEF	S1	S2	S3	S4	S5	S6	S7	S8
NaP	0.001	0.00335	0.00518	0.00709	0.00243	0.0004	0.00016	0.000153	0.00016
Acy	0.001	0.00665	0.0103	0.0201	0.00222	0.00088	0.00058	0.00012	0.00034
Ace	0.001	0.0077	0.00273	0.0034	0.00017	0.0006	0.0002	0	0.0001

Flu	0.001	0.0087	0.011	0.0108	0.00059	0.00073	0.00026	0.00012	0.00017
Phe	0.001	0.0311	0.0414	0.0598	0.004	0.00233	0.00177	0.000433	0.00187
Ant	0.01	0.09	0.162	0.274	0.0163	0.009	0.0073	0.001	0.0043
Flua	0.001	0.0282	0.0446	0.0684	0.00853	0.0022	0.00253	0.0006	0.00217
Pyr	0.001	0.0239	0.0389	0.0623	0.0083	0.00247	0.0026	0.0005	0.00187
BaA	0.1	1.19	2.25	4.6	0.487	0.167	0.163	0.0233	0.097
Chr	0.001	0.0107	0.0195	0.0433	0.0049	0.00167	0.00163	0.0003	0.0011
BbF	0.1	1.9	3.49	7.54	1	0.273	0.267	0.0467	0.15
BkF	0.1	0.727	1.35	2.87	0.367	0.1	0.1	0.02	0.06
BaP	1	13.3	25.2	51.1	6.6	1.73	1.77	0.267	1
IcdP	0.1	0.62	1.12	2.43	0.387	0.067	0.077	0.015	0.06
DahA	1	1.6	2.73	6.47	0.87	0.23	0.2	0	0.15
BghiP	0.01	0.053	0.0923	0.195	0.0373	0.006	0.0077	0.0015	0.006
∑16PAHs		19.6003	36.56791	75.75419	9.79574	2.59328	2.60173	0.376726	1.53508
